# Role of Cation Structure in CO_2_ Separation by Ionic Liquid/Sulfonated Polyimide Composite Membrane

**DOI:** 10.3390/membranes9070081

**Published:** 2019-07-04

**Authors:** Eri Hayashi, Kei Hashimoto, Morgan L. Thomas, Seiji Tsuzuki, Masayoshi Watanabe

**Affiliations:** 1 Department of Chemistry and Biotechnology, Yokohama National University, 79-5 Tokiwadai, Hodogaya-ku, Yokohama 240-8501, Japan; 2 Research Center for Computational Design of Advanced Functional Materials (CD-FMat), National Institute of Advanced Industrial Science and Technology (AIST), Tsukuba Central 2, 1-1-1 Umezono, Tsukuba, Ibaraki 305-8568, Japan

**Keywords:** carbon dioxide separation membrane, protic ionic liquid, sulfonated polyimide

## Abstract

The development of suitable separation technologies for the separation of carbon dioxide is a pressing technological requirement. The application of ion gel membranes for this purpose continues to stimulate a great deal of research, and in this study we focus on the chemical structure of the ionic liquid component in the ion gel, and its interactions with the sulfonated polyimide polymer. Whilst such membranes are known to give promising carbon dioxide separation properties together with mechanical strength and thin-film-processability, we further elaborate on how changing the cation of the ionic liquid from a typical imidazolium cation to a protic variant effects the physicochemical, thermal, and structural properties of the membranes, and how these changes further influence the carbon dioxide separation properties. We compare and contrast our findings with our earlier study on protic and aprotic ammonium-based ionic liquids, and highlight that for CO_2_ absorption behavior in the imidazolium systems, the importance of directionality of interactions (ion pairs exhibit a large energy stabilization only for a specific geometrical arrangement of cation and anion, e.g., hydrogen bonding rather than Coulombic interaction) between cation and anion applies not only to the protic system, but also to the nominally aprotic cation. Finally, we demonstrate that the phase separation behavior in the ion gels is an important factor in determining the carbon dioxide separation behavior.

## 1. Introduction

Ionic liquids (ILs), i.e., salts with a low melting point (<100 °C), have drawn considerable attention as a new class of solvents due to their unique characteristics, e.g., negligible vapor pressure, high chemical and thermal stability, and tunable features achieved through the combination of appropriate cations and anions [1,2,3]. The carbon dioxide (CO_2_) absorption capability of ILs is one of the most studied topics due to the emerging demand for the reduction of CO_2_ emissions, which is recognized as a crucial issue [4,5]. Membrane separation techniques using a dense membrane with gas permeability and CO_2_ selectivity, have many advantages for establishing a sustainable CO_2_ separation system with a low energy cost due to the simple driving force for separation [6,7,8,9,10]. The diffusion of CO_2_ is driven only by the partial pressure difference of CO_2_ gas across the membrane, and ideally only CO_2_ diffuses due to the high CO_2_ absorption selectivity of the membrane. By using ILs, we can bestow high CO_2_/N_2_ selectivity, while maintaining relatively good thermal stability, both intrinsic to many conventional ILs, to the separation membranes.

**Figure 1:**
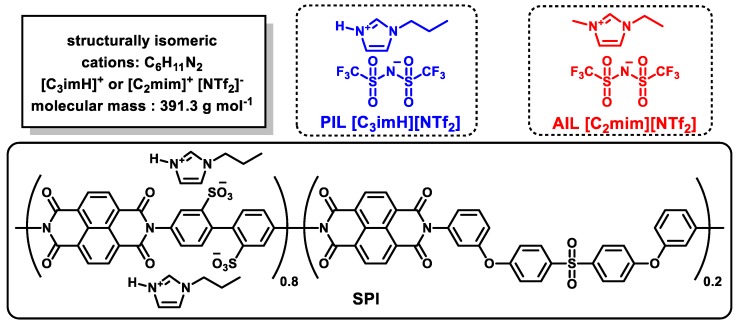
Chemical structures of protic ionic liquid (PIL), aprotic ionic liquid (AIL), and sulfonated polyimide (SPI) used in this study.

Ion gels, i.e., soft materials composed of a polymer network and IL, have been applied to CO_2_ separation membranes because the structure results in the strong retention of ILs in the polymer network without impairing the gas diffusivity [11]. Generally, a thin and tough membrane is required for CO_2_ separation to increase the permeation rate of CO_2_ gas, while preventing breakage of the thin membrane. We have reported that thin and tough composite membranes can be obtained using a sulfonated polyimide (SPI) as a polymer network [12,13]. These membranes exhibit excellent mechanical properties (elastic modulus > 10 MPa) even with a high loading of IL (IL content > 75 wt%), which can be ascribed to unique microstructures; the IL is mainly localized in the ionic domain of SPI (see Figure 1), and a nano-phase separated nonionic domain behaves as a mechanically tough polymer matrix [14]. Recently, we applied ammonium-type protic ionic liquids (PILs), a class of ILs with an active proton in the cation, to the IL/SPI composite membrane [15]. Such PILs are typically prepared by the simple mixing of the appropriate stoichiometric quantities of a suitable acid and suitable base, where-upon proton transfer occurs, and the resulting conjugate base (of the acid) and conjugate acid (of the base) then comprising the anion and cation of the IL, respectively. Generally, PILs exhibit low thermal stability; neutral acid and base, which easily evaporate and are corrosive in certain cases, are formed by the self-dissociation reaction in PILs. The self-dissociation reaction of PILs (or in other words, incomplete proton transfer from acid to base) is governed by the difference of acid dissociation constant between the acid and the protonated base (Δp*K*_a_) [16], and, thus, PILs with a high Δp*K*_a_ (typically more than 15, although lower in some cases) contain negligible amounts of neutral acid and base [17,18,19]. Thus, an ammonium-type PIL/SPI composite membrane containing a PIL with a high Δp*K*_a_ exhibited high thermal stability, which can be applicable to a CO_2_ separation membrane. Although the ammonium-type PIL/SPI composite membrane exhibited good CO_2_ permeability and selectivity comparable with an analogous aprotic ionic liquid (AIL)/SPI composite membrane (with an aprotic isomer of the PIL cation as cation), the effect of the active proton in the cationic structure on the CO_2_ separation properties is still unclear at the present stage.

In this work, to further clarify the role of the active proton of a PIL on CO_2_ absorption/separation, we applied an imidazolium-type PIL to an IL/SPI composite membrane, and compared the CO_2_ absorption/separation properties with a corresponding isomeric AIL/SPI membrane. With respect to the difference between imidazolium-type ILs and ammonium-type ILs, we further elaborate on the effect of the chemical structure of the cation on the separation properties of IL/SPI composite membranes. We also highlight the importance of directionality of interactions in the imidazolium systems in the CO_2_ absorption behavior. Here, directionality involves consideration of hydrogen bonding with strong directionality, as opposed to non-directionality of Coulombic interactions. 

## 2. Materials and Methods 

### 2.1. Materials

1-Ethyl-3-methylimidazolium bis(trifluoromethanesulfonyl)amide ([C_2_mim][NTf_2_]; Tokyo Chemical Industry Co., Japan), benzoic acid (Wako Chemicals, Osaka, Japan), and bis(trifluoromethanesulfonyl)amide acid (HNTf_2_; Kanto Chemical, Japan)) were used as received. 1-propylimidazole (C_3_im; Tokyo Chemical Industry Co., Japan) and *m*-cresol (Kanto Chemical, Hiratsuka, Kanagawa, Japan) were distilled prior to use to eliminate impurities. Bis[4-(3-aminophenoxy)-phenyl]sulfone (3BAPPS; Tokyo Chemical Industry Co., Tokyo, Japan) was recrystallized with an ethanol/water mixture prior to use. 1,4,5,8-Naphthalene-tetracarboxylic dianhydride (NTDA; Sigma-Aldrich, St. Louis, MO, USA) was dispersed in dimethylformamide (DMF), followed by refluxing at 60 °C for 1 day to remove impurities miscible in DMF. The resulting solid was washed several times with acetone and vacuum-dried. 2,2-Benzidinedisulfonic acid (BDSA; Tokyo Chemical Industry Co., Tokyo, Japan) was dissolved in aqueous solution with an excess amount of triethylamine. The resulting solid impurities were removed by filtration. 1 M H_2_SO_4_ aqueous solution was successively added to the solution and pure BDSA precipitated as a white solid. The obtained white solid was washed several times with water and vacuum-dried.

### 2.2. Preparation of Protic Ionic Liquid, Sulfonated Polyimide, and Composite Membranes

1-Propylimidazolium bis(trifluoromethanesulfonyl)amide ([C_3_imH][NTf_2_]) was prepared by the neutralization of equimolar amounts of the Brønsted acid, HNTf_2_ and Brønsted base, C_3_im, according to the reported method [20]. After the reaction, the obtained liquid was vacuum-dried at 80 °C for 48 h. It was confirmed that the water content was <20 ppm by Karl-Fischer titration.

[C_3_imH]-type-SPI was synthesized in two reaction steps (polyaddition and the chemical imidization reaction) in *m*-cresol according to the previously reported method [12]. BDSA and 3BAPPS (BDSA:3BAPPS = 4:1) and the equivalent amounts of NTDA were employed. The molecular weight of the SPI (*M*_n_ = 3.5 × 10^2^ kDa; *M*_w_ = 8.5 × 10^2^ kDa; *M*_w_/*M*_n_ = 2.4) was estimated by gel permeation chromatography (GPC, standard: polystyrene) on a Shimazu LC-20 series, with a Tosoh TSKgel G3000Hxl column (temperature: 40 °C; detector: UV-vis detector; volume of injection loop: 100 μL; mobile phase: DMF). Chemical structures of the PIL, AIL, and SPI are shown in Figure 1.

All [C_3_imH][NTf_2_]/SPI, and [C_2_mim][NTf_2_]/SPI composite membranes (IL content: 75 wt%) were prepared by a solution casting method using *m*-cresol as a co-solvent according to the previously reported method [12]. Transparent, uniform, and flexible membranes, for which no leakage of ILs was observed, were obtained. 

### 2.3. Gas Permeability Measurement

CO_2_ and N_2_ permeabilities were measured with a gas permeation measurement apparatus, GTR-10XFKS (GTR-Tech, Uji, Kyoto, Japan), according to our previous report [15]. A sketch of the experimental setup is shown in Figure S1. Helium gas was used as the carrier gas. The membranes were placed at the center of the permeation cell (permeation area was 9.62 cm^2^), and the test gas (either CO_2_ or N_2_) was introduced to one side of the membrane at a flow rate of 30 cm^3^ min^−1^ while the carrier gas was introduced to the other side of the membrane at a flow rate of 50 cm^3^ min^−1^. The flow of gases was controlled with digital mass flow controllers (KOFLOC, Kyotanabe, Kyoto, Japan) and additionally measured using a HORIBA film flow meter. The partial pressures of the permeated gases were obtained from the concentration of gases quantified by a gas chromatograph, Yanaco G2700T with a thermal conductivity detector (Yanaco Technical Science, Tokyo, Japan), pre-calibrated by measurement of a known concentration of the gas. A pressure of carrier gas and a total pressure of test gas were set at 76 cm Hg (atmospheric pressure). The measurements were conducted five times for a single membrane to estimate gas permeability, and the obtained values were averaged. The gas permeability coefficients, *P*_CO2_ and *P*_N2_ (Barrer = 1 × 10^−10^ cm^3^ (STP) cm cm^−2^ s ^−1^ (cm Hg)^−1^), were calculated using the following equation:

(1) $$P\;\left[\mathrm{Barrer}\right]=\frac{Q\;[{\mathrm{cm}}^{3}]\times X\;[\mathrm{cm}]}{A\;[{\mathrm{cm}}^{2}]\times t\;[s]\times C\;[\mathrm{cm}\;\mathrm{Hg}]}\times \frac{273.15\;[K]}{T\;[K]}\times 10^{10}$$

where *Q* [cm^3^] is the volume of the test gas permeated across the membrane, *X* [cm] is the sample thickness, *A* [cm^2^] is the permeation area, *t* [s] is the measuring time, *C* [cm Hg] is the partial pressure of the test gas, and *T* [K] is the absolute temperature. The measurement was carried out at 30 °C. Membranes used for the gas permeability measurements were ca. 90 μm in thickness.

### 2.4. Volume Expansion and CO_2_ Solubility Measurements for the Ionic Liquids

The volume expansion measurement was performed at 30 °C according to the previously reported procedure [15]. The sample was poured into a sapphire tube in an Ar-filled glovebox. Successively, the tube and apparatus were evacuated. Pressurized CO_2_ was introduced to the tube containing samples, and the volume of the sample was calculated from the height of the sample. The stabilized CO_2_ pressure (*p*) was measured with a pressure sensor PACE1000 (GE Druck, Surrey, UK). The volume expansion coefficient (Δ*V^L^*(*p*)) can be defined as the following equation.

(2) $$\Delta V^{L}\left(p\right)=\frac{V^{L}(p)\;[{\mathrm{cm}}^{3}]-V^{L}(p^{0})\;[{\mathrm{cm}}^{3}]}{V^{L}(p^{0})\;[{\mathrm{cm}}^{3}]}$$

where *V^L^*(*p*^0^) is the initial volume of the sample and *V^L^*(*p*) is the volume of the sample at *p*. We applied a polynomial fitting to Δ*V^L^*(*p*) vs. *p* plot to obtain the Δ*V^L^*(*p*) values at each experimental pressure.

The CO_2_ solubility measurement was performed at 30 °C according to the previously reported procedure [15]. The sample was poured into a high-pressure cell in an Ar-filled glovebox. After evacuation, a known amount of the pressurized CO_2_ was introduced to the cell containing the sample. The molar amount of CO_2_ in the liquid phase (*n^L^*(*p*)) can be obtained as follows;

(3) $$n^{L}\left(p)\;[\mathrm{mol}\right]=n^{i}\;[\mathrm{mol}]-\frac{V_{A}\;[{\mathrm{cm}}^{3}]-V^{L}(p)\;[{\mathrm{cm}}^{3}]}{V_{m}{}^{G}(p)\;[{\mathrm{cm}}^{3}{\;\mathrm{mol}}^{-1}]}$$

where *n^i^* is the molar amount of CO_2_ introduced to the apparatus, *V*_A_ is the total volume of the apparatus, and *V*_m_*^G^*(*p*) is the molar volume of the gas at 30 °C. The calibration procedure for determining the volume of the cell, *V*_A_, involves, at a constant temperature, introducing CO_2_ into the evacuated cell from a second vessel for which the volume, pressure, and mass of CO_2_ are known. The pressure change is then used to derive the volume of the cell using the equation of state [15]. Here, it is assumed that IL is not present in the gas phase due to the negligible vapor pressure of IL and negligible solubility of the IL in CO_2_, and, thus, *V*_m_*^G^*(*p*) corresponds to the molar volume of pure CO_2_. Using these values, the CO_2_ molar fraction (*x*_CO2_(*p*)) and solubility (*c*_CO2_(*p*)), and densities of the liquid phase (*ρ*^L^, IL) were calculated. 

### 2.5. Ab Initio Calculation

The Gaussian 09 program [21] was used for the ab initio calculations. The geometries of ion pairs and complexes were optimized at the HF/6-311G** level. The interaction energies were calculated at the MP2/6-311G** level. For the calculation of the intermolecular interaction energies (*E*_int_), the supermolecule method was used [22,23]. The basis set superposition error (BSSE) was corrected using the counterpoise method [24,25]. When anions form an ion pair, the energy of the anions increases due to the deformation. Thus, this deformation energy (*E*_def_) was taken account into the stabilization energy for the formation of ion pairs from isolated ions (*E*_form_), and *E*_form_ was calculated from the sum of *E*_int_ and *E*_def_. 

### 2.6. Other Measurements

Thermogravimetric analysis of the composite membranes was performed using a Seiko Instruments TG-DTA 7200C (Hitachi High-Technologies, Tokyo, Japan). Aluminum pans were used for the measurements. Then, the samples were first dried by holding at 100 °C for 30 min under N_2_ gas flow. After cooling to room temperature, thermogravimetric curves from 25 °C to 550 °C at a heating rate of 10 °C min^−1^ were recorded.

Differential scanning calorimetry (DSC) measurements were carried out using a Hitachi High-Tech DSC 6220 (Hitachi High-Technologies, Tokyo, Japan). For the hygroscopic IL samples, ILs were sealed in aluminum pans in a glove box filled with Ar ([H_2_O] <1 ppm.). For the other samples (SPI and membranes), the sealed sample pans were prepared in air. The cells were attached to the DSC apparatus, and heated to 150 °C, cooled to −150 °C and then heated again to 150 °C under N_2_ gas flow. The cooling and heating rates were 10 °C min^−1^. The DSC curves were recorded during the final reheating scans.

Tensile tests were performed with a Shimadzu EZ-LX 10N (Shimadzu, Kyoto, Japan). Samples were cut into dumbbell shapes (JIS K6251, 35 × 6 mm^2^ (total) and 10 × 2 mm^2^ (test area)) using a dumbbell cutter (Koubunshi Keiki, Kyoto, Japan). Stress–strain curves were measured by detecting the stress during the stretching of the samples at a speed of 1 mm s^−1^. Membranes used for the tensile measurements were of ca. 90 μm thickness.

Dynamic mechanical analysis (DMA) was performed for the membranes in a rectangular shape (6 mm × 10 mm) using a DMS7100 (Hitachi High-Tech Science, Tokyo, Japan). The sample was cooled to −150 °C, held for 30 min, and then heated to 250 °C at 2 °C min^−1^ under a N_2_ atmosphere. 

Measurements were performed at frequencies of 1, 2, 5, 10, and 20 Hz with a strain of 5 μm. Membranes used for the DMA measurements were of ca. 100 μm thickness. 

The density of [C_3_imH][NTf_2_] at 30 °C was measured using a thermoregulated density/specific gravity meter DA-100 (Kyoto Electronics Manufacturing Co. Ltd., Kyoto, Japan). The concentrations were derived from the densities and relative molecular masses.

## 3. Results and Discussions

### 3.1. Thermal Analyses

**Figure 2:**
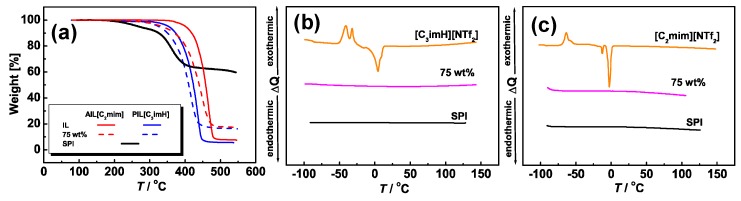
Thermogravimetric curves for ion gels containing 75 wt% of PIL[C_3_imH][NTf_2_] and AIL[C_2_mim][NTf_2_], together with neat SPI, PIL, and AIL. DSC thermograms of IL, ion gel, and SPI for (**b**) PIL[C_3_imH][NTf_2_] and (**c**) AIL[C_2_mim][NTf_2_].

First, we confirmed the thermal stability of the composite membranes. Figure 2 shows the Thermo gravimetric (TG) curves for SPI, PIL [C_3_imH][NTf_2_], AIL [C_2_mim][NTf_2_], and their ion gels. The thermal decomposition temperatures (*T*_d_), defined here as the temperature at 5% weight loss, are listed in Table S1. The initial weight loss of the pristine SPI in the range 200–300 °C occurs by the decomposition of sulfonated groups attached on the aromatic rings. On the other hand, the AIL and PIL exhibit higher thermal stability (decomposition starts from 300–400 °C), compared with that of the pristine SPI. PIL[C_3_imH][NTf_2_] showed high thermal stability (*T*_d_ ~350 °C), which can be ascribed to the large Δp*K*_a_ of the PIL (see Introduction), where the amount of free acid and base are negligible, analogous to our previous reports [18]. Interestingly, the composite membranes exhibit higher thermal stability than the pristine SPI, and the decomposition appears to be a single step. The exact reason for this phenomenon is not yet fully understood, but a similar phenomenon was observed in poly(methyl methacrylate)/AIL composite membranes [26]. The weight loss observed for the composite membranes in the range 300–500 °C can be attributed to the combined decomposition of both the IL (AIL or PIL) and SPI. The lack of clear (or step-wise) features corresponding to SPI in the TG curves for the composites is attributed to the higher proportion (i.e., 75 wt%) of the ILs. We also note here that it has been speculated that interactions between ILs and sulfonic acid groups of a polyelectrolyte may lead to improved thermal stabilities [27]. As a result, the composite membranes exhibit good thermal stability (*T*_d_ > 300 °C).

Figure 2b,c shows the DSC curves for the SPI, [C_3_imH][NTf_2_], [C_2_mim][NTf_2_], and their ion gels. Melting temperatures (*T*_m_) and glass transition temperatures (*T*_g_) of PIL and AIL are listed in Table S2. The peaks derived from *T*_m_ of the ILs were not observed in the composite membranes, indicating that SPI/IL membranes without exuding of ILs were successfully obtained. We previously reported that ILs are trapped in the nanospace formed by the ionic domains of the SPI, which results in the disappearance of the *T*_m_ of ILs [14,28]. Thus, this result implies that similar phase-separation structures between ionic and non-ionic domains were also formed in these membranes. Therefore, we concluded that thermally stable IL/SPI membranes were successfully obtained.

### 3.2. Gas Permeability Measurement

**Figure 3:**
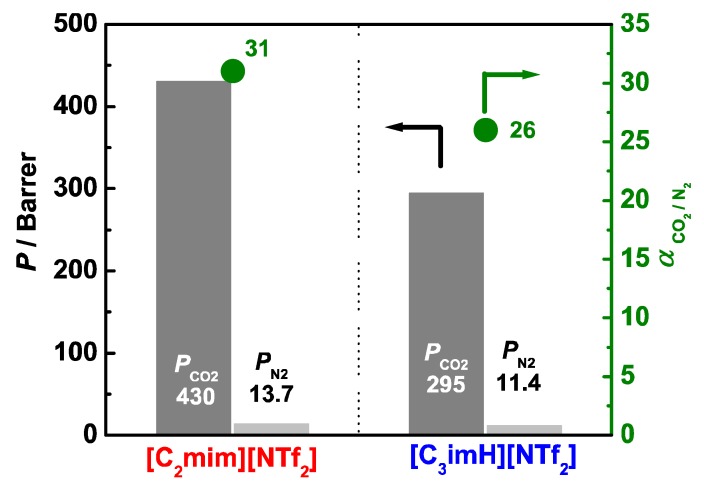
Permeability coefficients for CO_2_ and N_2_ gases (*P*_CO2_ and *P*_N2_, respectively), and CO_2_ selectivity (α) for AIL [C_2_mim][NTf_2_]/SPI and PIL [C_3_imH][NTf_2_]/SPI composite membranes.

The results of the gas permeability tests for [C_3_imH][NTf_2_]/SPI composite membrane and [C_2_mim][NTf_2_]/SPI composite membrane at 30 °C are shown in Figure 3 and Table S3. Although it was reported that pristine SPI membrane exhibited low gas permeability (*P*_CO2_ ~ 0.58) due to the high barrier property of SPI [28], IL/SPI composite membranes containing 75 wt% of IL exhibited good gas permeability. The AIL/SPI composite membrane exhibited a significantly higher permeability coefficient (*P*_CO2_ = 430 Barrer) and selectivity (α = 31) than those of the PIL/SPI composite membrane (*P*_CO2_ = 295 Barrer and α = 26) although the structural difference is quite small in these isomers. We previously reported that *P*_CO2_ of an ammonium-type PIL/SPI composite membrane (IL content: 75 wt%) exhibited slightly higher *P*_CO2_ (240 Barrer) than that of AIL (227 Barrer) while its α (~23) was lower than that of AIL (α ~ 26) [15]. In the ammonium-type system, the differences between PIL and AIL were small, but they could be ascribed to the slightly higher CO_2_ solubility of PIL at high CO_2_ pressure, and the slightly high plasticization effect of PIL on the hard non-ionic domain, which can form an extra gas permeation path. Thus, the conversely larger *P*_CO2_ value of AIL than PIL in the imidazolium system might be ascribed to the specific structural characteristics of the imidazolium-type cation (vide infra). The permeation coefficient of dense and non-porous membranes is determined by *P* = *S* × *D*, where *S* is the solubility of a permeate and *D* is the gas diffusivity. To elucidate which factor is governing the superior CO_2_ separation properties of the AIL/SPI composite membrane, we investigated CO_2_ solubility, *S*, of the ILs.

### 3.3. CO_2_ Absorption Behavior

**Figure 4:**
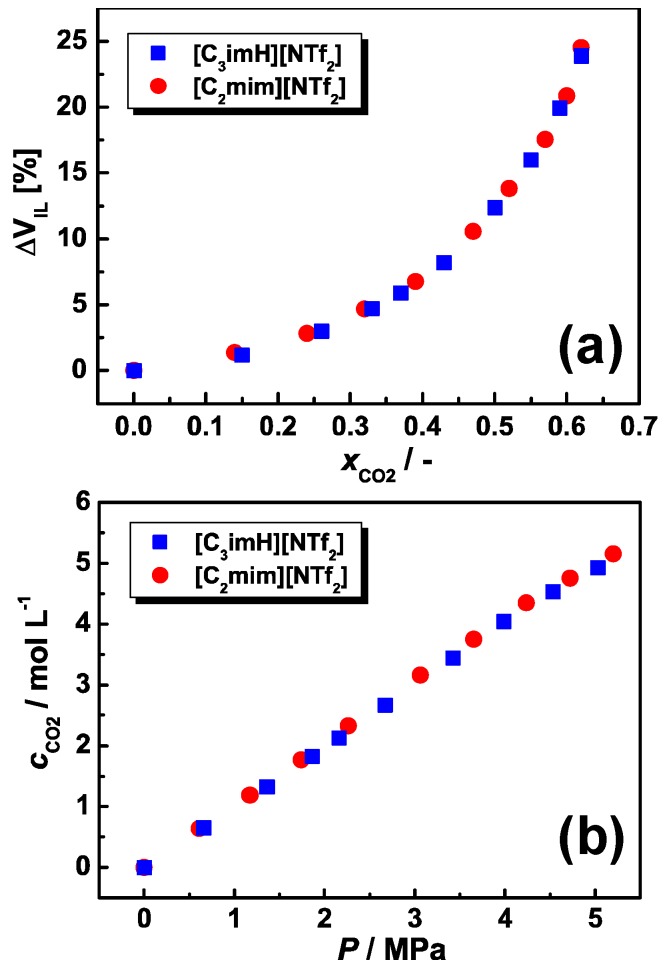
(**a**) Volume expansion of IL as a function of mole fraction of dissolved CO_2_ and (**b**) molarities of dissolved CO_2_ as a function of the pressure of CO_2_ for PIL [C_3_imH][NTf_2_] and AIL [C_2_mim][NTf_2_] at 30 °C.

Figure 4a,b shows volumetric expansion and CO_2_ solubility, respectively, for the AIL and PIL. The tabulated results were listed in Tables S4 and S5. A small volume expansion (<25% at CO_2_ mole fraction of 0.6) and good CO_2_ solubility, analogous to typical ILs, were observed [29]. The volumetric expansion and CO_2_ solubility for the AIL was of the same magnitude as those of the PIL, indicating that the difference of the CO_2_ permeability does not originate from the solubility. Note that, in the previously reported ammonium-type IL system, the volumetric expansion of the ammonium-type PIL at high pressure is obviously larger than the analogous ammonium-type AIL [15], whereas, in these imidazolium-type ILs, the AIL [C_2_mim][NTf_2_] showed the same volumetric expansion as the PIL [C_3_imH][NTf_2_] (~23%). This also resulted in slightly higher CO_2_ solubility for the ammonium-type PIL than that of the ammonium-type AIL [15], while the imidazolium-type PIL exhibited the same CO_2_ solubility as the imidazolium-type AIL.

**Figure 5:**
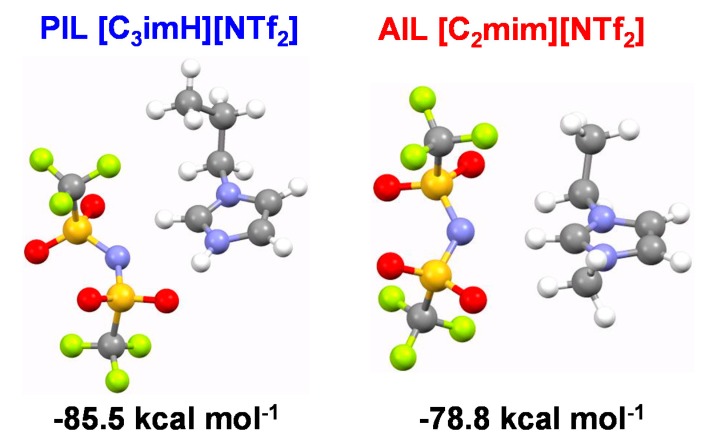
The most stable structures of [C_3_imH][NTf_2_], and [C_2_mim][NTf_2_] ion pairs, and their stabilization energies. Geometries were optimized at HF/6-311G** level. Stabilization energies were calculated at MP2/6-311G** level using the optimized geometries. Energy in kcal/mol.

From the viewpoint of structural change when ILs absorb CO_2_, the intermolecular interaction between CO_2_–cation, CO_2_–anion, and cation–anion is a key factor to understand the different CO_2_ absorption behaviors between the imidazolium and ammonium systems. Therefore, ab initio calculation was performed to obtain further information on the interaction between CO_2_ and ion pairs. Figure S2 shows the most stable structures and the stabilization energy of CO_2_–[C_3_imH]^+^ (cation of PIL), CO_2_–[C_2_mim]^+^ (cation of AIL), and CO_2_–[NTf_2_]^−^ (anion) complexes. The stabilization energies are negative but small, indicating that there is no strong attractive interaction between the IL components and CO_2_. The magnitude of the stabilization energy of CO_2_–[C_3_imH]^+^ is slightly larger than that of the other components. Moreover, CO_2_ preferentially interacts with the NH bonds of the PIL cation. This is likely to be due to the interaction between the active protons and the negatively charged O atom of CO_2_. We also evaluated the stabilization energies of anion–cation complexes (Figure 5). The stabilization energy of the ion pair (< −70 kcal mol^−1^) is significantly larger than those of the CO_2_–cation and CO_2_–anion complexes (~ −5 kcal mol^−1^). This result indicates that dissolution of CO_2_ leads to a loss of stabilization energy, which is consistent with the fact that pressurization of CO_2_ is necessary to dissolve CO_2_ into the IL.

Here, we also note that in the previously reported ammonium-type IL, strong directional interaction is significant only when active protons of PIL are present (PIL: –90.9 kcal mol^−1^, AIL: –77.6 kcal mol^−1^) [15]. Thus, we concluded in that study that the difference in larger volumetric expansion and CO_2_ absorption could be ascribed to the formation of ion pairs in the PIL, which results in a larger space for CO_2_ absorption in the voids between ion pairs. In contrast, the difference of stabilization energy between PIL [C_3_imH][NTf_2_] (–85.5 kcal mol^−1^) and AIL [C_2_mim][NTf_2_] (–78.8 kcal mol^−1^) was 6.7 kcal mol^−1^ in imidazolium-type ILs in the current study (Figure 5), which is smaller than that (13.3 kcal mol^−1^) of the ammonium-type IL system. Moreover, the directionality of interaction between cation and anion was observed in both PIL and AIL; the O atom of the NTf_2_ anion orientates towards the NH bond of the C_3_imH cation, and the N atom of the NTf_2_ anion towards the CH bond at C2 position of the C_2_mim cation. The directionality of the interaction for the C_2_mim cation has been attributed to the partial positive charge at the C2 proton, resulting in hydrogen bonding between the cation CH bond and anion [30]. Thus, it is also plausible (or perhaps self-evident) that the NH of the PIL cation, with an active proton, may engage in a hydrogen bonding interaction with the anion. Thereby, directionality of interaction exists in both AIL and PIL for these imidazolium-type ILs, which might lead to the smaller difference in the degree of ion pair formation. Therefore, the presence or absence of active protons does not significantly influence the degree of ion pair formation, resulting in a smaller difference in the CO_2_ absorption behavior between AIL and PIL in imidazolium-type ILs compared to the ammonium-type ILs.

### 3.4. Plasticization Effect by IL

From the solubility test, PIL and AIL showed equivalent solubility, implying that the difference of CO_2_ permeability could be due to the difference in gas diffusion in the membrane. In IL/SPI composite membranes, the formation of a gas permeation path is a key factor to enable good CO_2_ permeability [14,28]; the SPI composite membrane has a phase separation structure with a hard domain composed of nonionic domain of SPI, and a soft domain composed of IL and ionic domain of SPI. The CO_2_ gas dissolves and diffuses mainly in the ionic domain, and, thus, the formation of a continuous phase of soft domain with increasing IL content leads to a significant increase of the CO_2_ permeability. We previously estimated the diffusion coefficient, *D*, of gases using a time-lag method and it increased with increasing IL content, while solubility, *S*, was almost constant in the analogous imidazolium-type AIL/SPI composite membranes [14]. This result strongly supports the idea that the change of the *D* is a key factor in the increase of CO_2_ permeability in IL/SPI composite membranes. Additionally, the plasticization of the hard domain also contributes to the increase of CO_2_ permeability; however, this also leads to a decrease of CO_2_ selectivity, indicating that the plasticization of the hard domain impairs the gas barrier properties of the SPI [15]. In the ammonium-type IL system, the larger plasticization effect of PIL on the nonionic domain of SPI was clearly observed, resulting in higher permeability and lower selectivity of CO_2_ than those of AIL. Therefore, in order to investigate the degree of the plasticization of the membrane by IL, tensile tests were performed.

**Figure 6:**
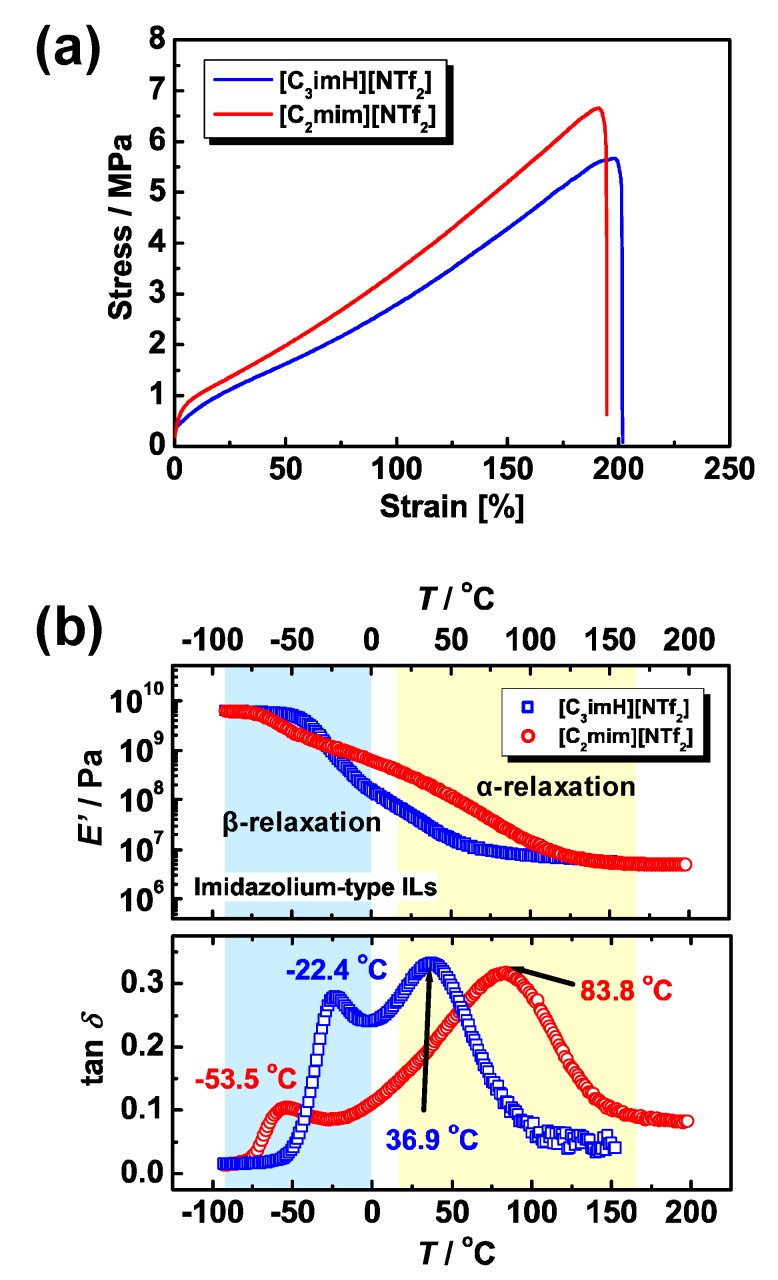
(**a**) Stress–strain curve and (**b**) results of dynamic mechanical analysis (DMA) analysis for PIL/SPI and AIL/SPI membranes containing 75 wt% of IL.

Figure 6a shows the stress–strain curves obtained from tensile tests for PIL [C_3_imH][NTf_2_]/SPI and AIL [C_2_mim][NTf_2_]/SPI composite membranes. The AIL membrane and the PIL membrane showed similar fracture strain, modulus of elasticity, and fracture energy, as listed in Table S6, where the PIL membrane gives a slightly softer material. In the ammonium-type IL system, the stronger plasticization effect of PIL on the membrane was observed, resulting in higher breaking strain (~300%) than that of the AIL (~200%), which can be ascribed to the affinity of the active proton with the nonionic part of SPI [15]. In the imidazolium-type IL system, the difference in the apparent plasticization effect on the membrane was not observed. To further elucidate the degree of the plasticization effect on the ionic and nonionic domain, we conducted dynamic viscoelasticity measurements. 

Figure 6b shows the results of DMA of SPI composite membranes containing PIL [C_3_imH][NTf_2_] and AIL [C_2_mim][NTf_2_]. Two peaks for tan δ (α relaxation: high-temperature side; β relaxation: low-temperature side) were observed in all the composite membranes, which can be assigned to the relaxations of the nonionic domain and ionic domain, respectively, according to the previous reports [14]. In the previous study, the neat SPI membrane exhibited two relaxations (*T*_α_ > 200 °C and *T*_β_ = 40.5 °C), and *T*_α_ and *T*_β_ rapidly decreased with increasing IL content [14]. In this study, the 75 wt% composite membranes exhibited lower *T*_α_ and *T*_β_ than those for neat SPI membrane, and, thus, we concluded that ionic/non-ionic domains are plasticized. In contrast to the tensile test, both the *T*_α_ and *T*_β_ of PIL/SPI were significantly different from those of AIL/SPI. *T*_α_ of the PIL membrane (36.9 °C) was lower than *T*_α_ of the AIL membrane (83.8 °C) in the imidazolium-type IL system, which is the same tendency as those of the ammonium-type IL system (*T*_α_ of PIL membrane: 60.2 °C; *T*_α_ of AIL membrane: 118.2 °C) [15]. Thus, the lower *T*_α_ for membranes with the imidazolium-type PIL than the corresponding AIL could be ascribed to the interaction between the positively charged active proton within the PIL and the nonionic portion, analogous to the ammonium-type IL system. On the other hand, *T*_β_ of the AIL membrane (−53.5 °C) was significantly lower than *T*_β_ of the PIL membrane (−22.4 °C) in the imidazolium-type IL system while the difference of *T*_β_ was small in the ammonium-type IL system (*T*_β_ of AIL membrane: −46.9 °C; *T*_β_ of PIL membrane: −38.8 °C) [15]. These results indicate that the ionic domain is selectively and strongly plasticized by AIL [C_2_mim] cation while both ionic and nonionic domains are weakly plasticized by PIL [C_3_imH] cation, which might be the reason why the plasticization effect of the whole membrane was of the same order in the tensile test (Figure 6a). In imidazolium-type PIL/SPI membranes, the temperature difference between *T*_α_ and *T*_β_ decreases with increasing the PIL content, indicating that the phase-separated structure becomes unclear. On the contrary, in imidazolium-type AIL/SPI membranes, the main CO_2_ permeation path formed by the ionic domain was strongly plasticized by IL. Moreover, the large difference between *T*_α_ and *T*_β_ indicates a more substantial phase separation between the ionic and nonionic domains. In the weakly phase-separated structure, both the ionic and non-ionic phases contain a high amount of polymer, and, thus, their microscopic viscosity could be significantly higher than that of neat IL. On the other hand, in the strongly phase-separated structure, the ionic domain is highly plasticized, and the viscosity of this part is presumed to be comparatively rather low, which can contribute to a higher diffusion rate. Therefore, we conclude that the formation of a distinct continuous gas permeation path induced by the selective plasticization by the IL is the reason why the imidazolium-type AIL/SPI membrane exhibited the best CO_2_ permeability in these membranes, without impairing CO_2_ selectivity. 

## 4. Conclusions

IL/SPI composite membrane was prepared using imidazolium-type AIL and PIL and applied to CO_2_ separation membranes. The PIL membrane exhibited good CO_2_ separation properties (*P*_CO2_ = 295 Barrer, α = 26), indicating the possibility for application to the CO_2_ separation membrane, but the AIL membrane exhibited significantly better CO_2_ separation properties (*P*_CO2_ = 430 Barrer, α = 31); in fact, the best CO_2_ separation properties of the IL/SPI composite membrane we so far reported. Compared with the previously reported various IL/polymer composite membranes [31], these values are not so high because they strongly depend on the separation properties of IL itself. However, high thermal stability, the tunable affinity of SPI to IL by changing the counter cation, and the high mechanical toughness of the membrane, which leads to the preparation of a thinner membrane, are advantageous for the CO_2_ separation membranes. The imidazolium-type PIL and AIL were equivalent in terms of the CO_2_ solubility and the volume expansion coefficient upon dissolving CO_2_. From the tensile test and DMA, it was found that phase-separated structures are formed in these composite membranes and that the degree of plasticization of ionic domains, forming the main gas permeation path, is largest in imidazolium-type AIL. This could result in the best performance of imidazolium-type AIL/SPI membrane as a CO_2_ separation membrane. Therefore, we can conclude that the CO_2_ separation properties of IL/SPI composite membranes are not governed by the solubility of CO_2_ in IL and the presence of an active proton; but rather the affinity of the cation to the ionic/nonionic domain strongly affects the properties. 

## Supplementary Materials

The following are available online at https://www.mdpi.com/2077-0375/9/7/81/s1, Figure S1: a sketch of the instrument for gas permeability measurements, Figure S2: The most stable structures of [C_3_imH]^+^–CO_2_, [C_2_mim] ^+^–CO_2_, and [NTf_2_]^−^–CO_2_ complexes and their stabilization energies. Tables S1 and S2: thermal properties, Table S3: gas permeability, Tables S4 and S5: volumetric expansion and CO_2_ solubility, and Table S6: mechanical properties.
