# Supplementary material for: Role of Cation Structure in CO2 Separation by Ionic Liquid/Sulfonated Polyimide Composite Membrane
Source: Membranes (Basel). 2019 Jul 4;9(7):81. doi: 10.3390/membranes9070081 (PMC6680971; doi:10.3390/membranes9070081)
Supplement: Supplementary file 1 [file membranes-09-00081-s001.pdf]

membranes

Supplementary Materials: Role of Cation Structure in CO_2 Separation by Ionic Liquid/Sulfonated Polyimide Composite Membrane

Eri Hayashi [1], Kei Hashimoto [1], Morgan L. Thomas [1,§], Seiji Tsuzuki,[2] and Masayoshi Watanabe [1,*]

[1] Department of Chemistry and Biotechnology, Yokohama National University, 79-5 Tokiwadai, Hodogaya-ku, Yokohama 240-8501, Japan
[2] Research Center for Computational Design of Advanced Functional Materials (CD-FMat), National Institute of Advanced Industrial Science and Technology (AIST), Tsukuba Central 2, 1-1-1 Umezono, Tsukuba, Ibaraki 305-8568, Japan
§ Present address: Department of Materials and Life Sciences, Faculty of Science and Technology, Sophia University, 7-1 Kioicho, Chiyoda-ku, Tokyo 102-8554, Japan
* Correspondence: mwatanab@ynu.ac.jp; Tel.: +81- 45-339-3955

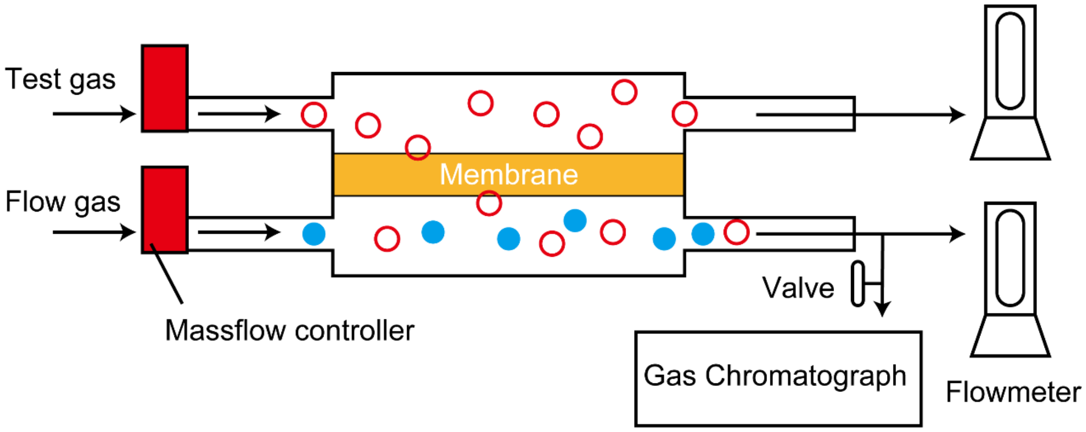

Figure S1. A sketch of gas permeation test.

Table S1. Decomposition temperatures of ion gels obtained by TG.

	**[C3imH][NTf2]** T_d / °C [a]	**[C2mim][NTf2]** T_d / °C [a]
IL	353.6	401.5
IL/SPI composite [b]	325.4	334.3
SPI	268.6	

[a] Decomposition temperature (T_d) is defined as the temperature at 5% weight loss.
[b] IL content of IL/SPI composite membrane is 75 wt%.

Table S2. Melting temperature (T_m) and glass transition temperature (T_g) for [C3imH][NTf2] and [C2mim][NTf2].

	[C3imH][NTf2]	[C2mim][NTf2][a]
T_m / ºC	4.8	−12.1, −2.7
T_g / ºC	−87.1	−94.0

Table S3 The results of gas permeability measurements performed under single gas conditions at 30 °C.[a]

	**[C_2mim][NTf_2]**	**[C_3imH][NTf_2]**
P_{CO2}	430	295
P_{N2}	13.7	11.4
α	31	26

[a]P_{CO2} / P_{N2}. Unit of gas permeability coefficient (P) is Barrer.

Table S4. CO_2 absorption properties of [C_3imH][NTf_2] at 30 °C.[a]

**P (MPa)**	x_{CO2}	c_{CO2} **(mol/l)**	ρ^L **(g/cm^3)**	**ΔV^L (%)**
0	0	0	1.49	0
0.66	0.15	0.65	1.50	1.16
1.36	0.26	1.32	1.50	2.97
1.86	0.33	1.82	1.50	4.68
2.16	0.37	2.13	1.50	5.88
2.67	0.43	2.67	1.49	8.21
3.42	0.50	3.44	1.48	12.35
3.99	0.55	4.04	1.46	15.98
4.53	0.59	4.53	1.44	19.91
5.03	0.62	4.93	1.42	23.90

[a] x_{CO2}, c_{CO2}, ρ^L and ΔV^L correspond to the mole fraction of CO_2, molarities of CO_2, densities and volume expansions of the liquid phase, respectively, in the CO_2/[C_3mimH][NTf_2] system.

Table S5. CO_2 absorption properties of [C_2mim][NTf_2] at 30 °C.[a]

**P (MPa)**	x_{CO2}	c_{CO2} **(mol/l)**	ρ^L **(g/cm^3)**	**ΔV^L (%)**
0	0	0	1.51	0
0.61	0.14	0.64	1.52	1.36
1.17	0.24	1.19	1.52	2.82
1.74	0.32	1.77	1.52	4.69
2.26	0.39	2.33	1.52	6.75
3.06	0.47	3.16	1.50	10.55
3.65	0.52	3.75	1.49	13.82
4.24	0.57	4.35	1.48	17.57
4.72	0.60	4.76	1.46	20.86
5.20	0.62	5.15	1.44	24.50

[a] x_{CO2}, c_{CO2}, ρ^L and ΔV^L correspond to the mole fraction of CO_2, molarities of CO_2, densities and volume expansions of the liquid phase, respectively, in the CO_2/[C_2mim][NTf_2] system.

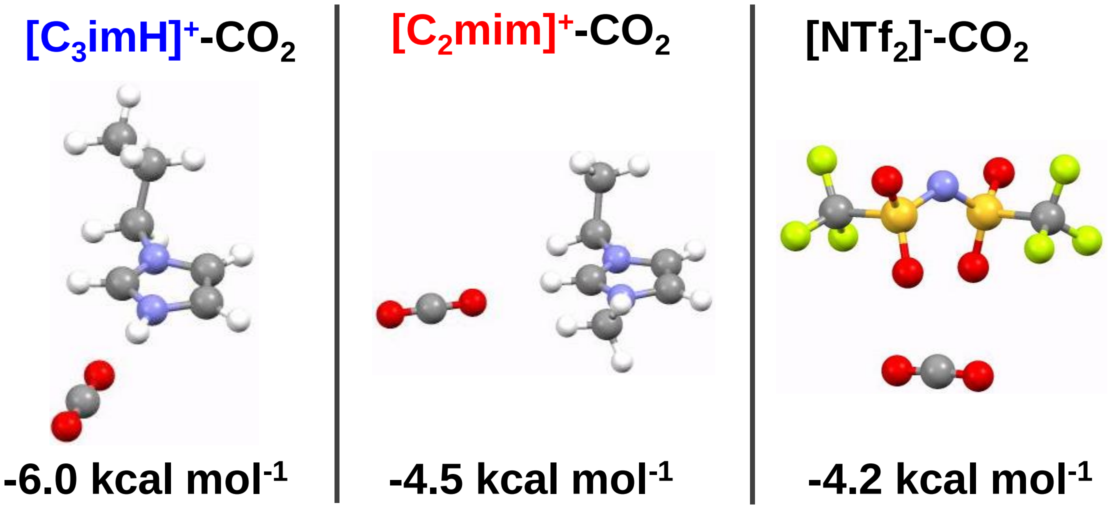

Figure S2. The most stable structures of $[C_3imH]^+–CO_2$, $[C_2mim]^+–CO_2$, and $[NTf_2]^-–CO_2$ complexes and their stabilization energies. Geometries were optimized at HF/6-311G** level. Stabilization energies were calculated at MP2/6-311G** level using the optimized geometries. Energy in kcal/mol.

Table S6 The elastic modulus (E), fracture stress (σ_f), fracture strain (λ_f), and fracture energy (W_f) of IL/SPI membranes.

**IL**[a]	***E*** **(MPa)**	**σ_f (MPa)**	**λ_f (%)**	**W_f (kJ/m^3)**
AIL	3.1	6.3	194	7098
PIL	2.6	5.3	201	6004

[a] AIL:$[C_2mim][NTf_2]$; PIL:$[C_3imH][NTf_2]$ (IL content : 75 wt%).

© 2019 by the authors. Submitted for possible open access publication under the terms and conditions of the Creative Commons Attribution (CC BY) license (http://creativecommons.org/licenses/by/4.0/).
